# Comparative analysis of sensory features, microbial diversity, and their correlations in light‐flavor Daqu from different regions

**DOI:** 10.1002/fsn3.4004

**Published:** 2024-02-06

**Authors:** Fanshu Xiang, Wenchao Cai, Zhuang Guo, Chunhui Shan

**Affiliations:** ^1^ School of Food Science Shihezi University Shihezi Xinjiang Autonomous Region China; ^2^ Hubei Provincial Engineering and Technology Research Center for Food Ingredients Hubei University of Arts and Science Xiangyang Hubei China; ^3^ Xiangyang Liquor Brewing Biotechnology and Application Enterprise‐University Joint Innovation Center Xiangyang Hubei China

**Keywords:** comparative analysis, different regions, electronic sensing technology, light‐flavor Daqu, microbial diversity

## Abstract

This study performed a comparative analysis of the sensory and microbial profiles of light‐flavor Bijou (LFD) from Taiyuan (Shanxi Province) and Suizhou (Hubei Province) in China. The results of the electronic nose showed that the aromatic substances of the LFD from Taiyuan (TLFD) were significantly higher (*p* < .05), while alcohol and aldehyde substances were significantly lower (*p* < .05) compared with the LFD from Suizhou (SLFD). The average response values of sensors W1C (sensitive to aromatic hydrocarbons), W3C (sensitive to amine and aromatic components), W5C (sensitive to olefins, aromatics, and polar molecules), and W2S (sensitive to alcohol and aldehyde compounds) to TLFD were 0.26, 0.33, 0.34, and 7.72, whereas the response values to SLFD were 0.25, 0.32, 0.33, and 8.04, respectively. The electronic tongue results showed that the aftertaste A (bitter aftertaste) and aftertaste B (astringent aftertaste) of the TLFD were significantly higher (*p* < .05) and umami was significantly lower (*p* < .05) as compared to the SLFD. The relative intensities of the aftertaste A, aftertaste B, and umami indicators of TLFD were 0.10, −0.008, and −0.22, respectively, while those of SLFD were −0.23, −0.36, and 0.835, respectively. MiSeq high‐throughput sequencing results showed that TLFD exhibited lower fungal richness and diversity compared to SLFD. The dominant bacterial genera were mainly *Bacillus* (58.12%), *Kroppenstedtia* (10.11%), and *Weissella* (6.26%), and the dominant fungal genera were *Saccharomycopsis* (67.53%), *Rasamsonia* (9.90%), and *Thermoascus* (7.10%). *Streptomyces* and *Staphylococcus* were identified as the key characteristic microorganisms in TLFD, while *Kroppenstedtia*, *Rasamsonia*, and *Thermoascus* were the key characteristic microorganisms in SLFD. Correlation analysis indicated a stronger correlation between microorganisms and sensory characteristics in SLFD samples. This study provides valuable insights into the sensory and microbiological characteristics of LFD from different regions and offers a new perspective for understanding the production of differently flavored light‐flavor Baijiu.

## Graphical abstract

The sensory characteristics of LFD in the two regions differed significantly. The higher fungal richness and diversity were found in LFD from the Suizhou region. *Bacillus*, *Kroppenstedtia*, *Saccharomycopsis*, and *Rasamsonia* were the dominant microbiota in LFD. The correlations between the microbiota and sensory characteristics of the LFD from the Suizhou region were stronger.

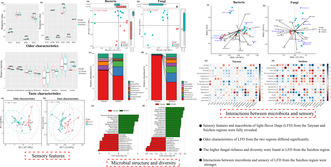

## 1 INTRODUCTION

Baijiu, a world‐renowned beverage, is among the oldest distilled liquors across the globe. Notably, it is also an extremely important part of the Chinese food and beverage industry (Hong et al., 2021). According to its flavor characteristics, Baijiu is divided into 12 types, including the light‐flavor type, strong‐flavor type, and sauce‐flavor type (Hong et al., 2020). Baijiu is typically brewed in a relatively open environment, and the microbial fermentation system required for Baijiu production consists of several raw materials, an adequate production environment, Qu, producers, etc. (Xu et al., 2017). The type of Qu (fermentation starter for Chinese liquors) and the brewing process vary depending on the flavor of Baijiu (Hong et al., 2021). The Qu is rich in microbes and enzymes, which are the main drivers of fermentation (Yang, Wang, et al., 2021). An old Chinese saying states that “the Qu is the bone of liquor,” which fully reflects the importance of the Qu in determining liquor quality (Gan et al., 2019).

**FIGURE 1:**
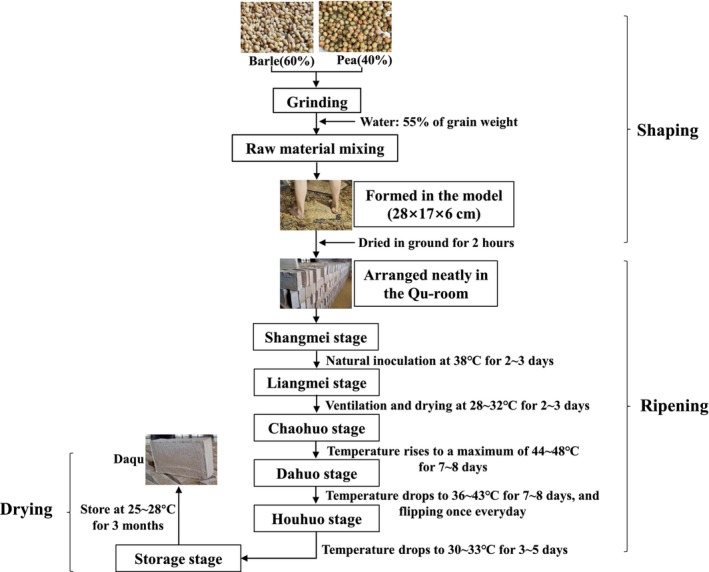
Process of light‐flavor Daqu production.

Light‐flavor Baijiu (LFB) is one of the three major liquors in China, and it is favored by many consumers for its clear, mellow, sweet, pure, and elegant flavor with a clean finish (Zheng & Han, 2016). Light‐flavor Daqu (LFD) is the saccharifying agent used for making LFB, and its influence on the flavor of the liquor cannot be ignored. The production of LFD is complicated and involves the following steps: (1) Raw material mixing: crushed wheat and peas mixed well with water; (2) Daqu formation: the raw material loaded into a Qu‐mold to make Daqu blocks; (3) Stacking: Daqu blocks are neatly arranged in the Qu‐room; (4) Fermentation: fermentation is further divided into six stages: Shangmei, Liangmei, Chaohuo, Dahuo, and Houhuo. During Shangmei, the microorganisms in the natural environment are inoculated onto the surface of Daqu blocks and begin to grow. During Liangmei, the room is ventilated and water is evaporated so that the moisture in the Daqu reaches equilibrium. Then, during Chaohuo, the room is gradually heated to 44–48°C, allowing microorganisms to enter and multiply inside the Daqu block. At the next stage, Dahuo, the temperature of the room slowly drops to about 40°C and the water evaporates, making the Daqu dry. Finally, during Houhuo, the temperature of the room drops to 33°C, and the temperature of the Daqu also drops gradually; (5) Removal from the Qu‐room: the Daqu is transferred from the Qu‐room to a dry and ventilated cool room and stored for 3 months (Figure 1; Hu et al., 2021). After fermentation, the LFD is enriched with hundreds of microorganisms, all of which play a crucial role in the production of liquor, including the induction of saccharification reactions and the synthesis of ethanol and various aroma substances (Zheng et al., 2014).

LFB originated in Shanxi Province, China, and was later introduced to Hubei Province, China, due to migration. However, the flavor of LFB continues to differ from one region to another due to the subtle differences in the LFD and production processes used. Owing to differences in microbial ecology and production processes, the microbial composition of Daqu is also significantly different across different regions (Wang et al., 2021). This phenomenon is also observed in several types of fermented foods, such as cheese and kimchi (Xiao et al., 2018; Yang et al., 2022; Yang, You, et al., 2021). Previous studies have focused more on the microbial taxa and functional differences in different types of LFD (Cai et al., 2021; Hou et al., 2022). Using metagenomic technology, Hou et al. found that different types of LFD shared a large number of microbial communities, but their microbial functions were complementary, collectively shaping the flavor quality of LFB (Hou et al., 2022). However, currently, the differences in the sensory features and microbial diversity of LFD from different regions are not well understood. It is meaningful to study the sensory qualities and microbial profiles of LFD from different production regions and also to analyze the connection between these factors. These can provide greater insights into the quality characteristics of LFD from different regions and the mechanisms of the production of different types of LFB.

Electronic sensing techniques, which involve the use of electronic nose and an electronic tongue devices, are gradually becoming more sophisticated (Cai et al., 2021). Unlike chromatographic techniques, they do not require complex sample preprocessing. Further, they greatly preserve the integrity of the sample, are more portable, and enable faster operation (Karakaya et al., 2020; Ross, 2021). Similarly, MiSeq second‐generation high‐throughput sequencing technology provides several advantages for the comprehensive and rapid evaluation of microbial composition (Sun et al., 2018; Zhu et al., 2022). Thus, it is valuable for the comparative analysis of microbial diversity between different groups of samples (Huang et al., 2019).

This study systematically investigated the differences in sensory features and microbial diversity of LFD produced in two different regions. Digital evaluation and comparative analysis of odor and taste indices in LFD samples were performed using an electronic nose and electronic tongue, respectively. Then, the differences in microbial community structure, characteristic microorganisms, and core microbial community were analyzed using second‐generation sequencing technology. Finally, the correlation between microorganisms and sensory indices was delineated. This study greatly enriches our knowledge of the sensory and microbial characteristics of LFD from different regions and provides a new perspective for understanding the synthesis of LFB with different flavors.

## 2 MATERIALS AND METHODS

### 2.1 Sample collection

In April 2022, 10 samples of Qingxiang Daqu were collected from both Taiyuan, Shanxi Province, and Suizhou, Hubei Province. Accordingly, a total of 20 samples were examined in this study. LFD from the Taiyuan and Suizhou regions were labeled TLFD and SLFD, respectively. The samples from the same region were produced by the same enterprise and were from the same batch. All samples were made from wheat and peas and fermented for 1 month and stored for 6 months.

### 2.2 Sample macrogenomic DNA extraction, PCR amplification, and sequencing

Macrogenomic DNA (deoxyribonucleic acid) was extracted using the Macrogenomic DNA Extraction Kit (D5625‐01, Omega Bio‐Tek Inc., USA). The primers 338F/806R (338F: 5′‐ACTCCTACGGGAGGCAGCA‐3′; 806R: 5′‐GGACTACHVGGGTWTCTAAT‐3′) and ITS3F/ITS4R (ITS3F: 5′‐GCATCGATGAAGAACGCAGC‐3′; ITS4R: 5’‐TCCTCCGCTTATTGATATGC‐3′) were used to perform PCR and amplify the 16S V_3_ ~ V_4_ region and ITS 2 region, respectively. The amplification procedure was based on the protocol described by Hu et al. (2020). PCR amplification products were quality tested, cleaned, and purified, and the purified PCR products were sent to Shanghai Meiji Biomedical Technology Co., Ltd. for sequencing on the Illumina MiSeq PE300 platform (Illumina, San Diego, CA, USA).

### 2.3 Bioinformatics analysis

Sequencing data were analyzed bioinformatically on the QIIME (v1.9.0) platform. First, PyNAST (v 1.2.2) software was used to split and merge the sequence data into individual samples based on nucleotide tag information. Then, double‐ended sequences were spliced using FLASH (v 1.2.7) software, and the sequences were quality controlled based on the criteria proposed by Caporaso et al. (2010). Valid sequences passing quality control criteria were used to classify operational taxonomic units (OTUs) based on 97% sequence similarity, and then the UCHIME software was used to identify and remove chimeras of OTU sequences (Edgar et al., 2011; Wei et al., 2021). The annotation of bacterial and fungal species was subsequently completed based on the RDP (v 11.6) and Unite (v 7.2) databases, respectively (Brandt et al., 2021; Nilsson et al., 2019). Meanwhile, α‐diversity indices such as the Chao1 index and Shannon index were calculated for the bacteria and fungi in each sample. The β‐diversity of LFD from different regions was evaluated based on Bray–Curtis, unweighted and weighted UniFrac distances, and principal coordinate analysis (PCoA). Finally, the LEfSe tool (http:// huttenhower.sph.harvard.edu/galaxy/) was applied to screen the characteristic microbial taxa for the LFD from different regions.

### 2.4 Odor analysis

The odor of LFD was identified using a PEN3 electronic nose instrument (AIRSENSE Analytics Co., St., Mecklenburg‐Vorpommern, Germany). Each sample was weighed (8.0 g), added to a headspace vial using a headspace injector, and placed at 45°C for 10 min. The injection conditions were set up according to the method described by Cai et al. (2020). Three assays were performed in parallel for each sample. Further, the average of the sensor response values at 49, 50, and 51 s was selected as the data for subsequent analysis. Correlations between microbial taxa and odor indicators were examined using redundancy analysis (RDA) and Pearson's test.

### 2.5 Taste analysis

The taste indices of LFD were examined using an SA402B electronic tongue instrument (Intelligent Sensor Technology, Inc., Tokyo, Japan). The samples were diluted at a sample‐to‐distilled water ratio of 1:4 (HTD sample:distilled water = 30.0 g:120 mL), and the well‐mixed dilutions were allowed to equilibrate at 25°C for 30 min. Subsequently, the dilutions were centrifuged at 4°C and 10,000 g for 10 min, and the supernatant was filtered through qualitative filter paper. Four assays were performed in parallel for each sample, and the average of the last three assays was taken as the data for subsequent analysis (Cai et al., 2020). Correlations between microbial taxa and taste indicators were also examined using RDA and Pearson's test.

## 3 RESULTS

### 3.1 Sensory quality analysis of LFD based on electronic‐sensing technology

#### 3.1.1 Aroma analysis

**FIGURE 2:**
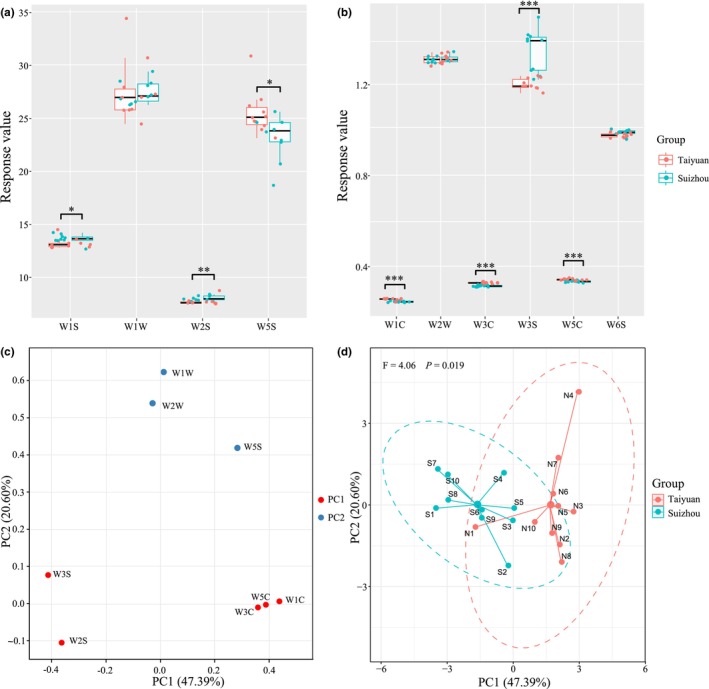
Odor analysis of light‐flavor Daqu based on electronic nose sensors (a, b). * indicates *p* < .05; ** indicates *p* < .01; *** indicates *p* < .001. Principal component analysis loading plot (c) and factor score plot (d) based on odor indicators. Sensor W1W is sensitive to terpenes and organic sulfur compounds; sensor W2W is sensitive to aromatic compounds and organic sulfur compounds; sensor W1S is sensitive to methyl compounds; sensor W2S is sensitive to alcohol and aldehyde/ketone compounds; sensor W3S is sensitive to alkanes; sensor W5S is sensitive to nitrogen compounds; sensor W6S is sensitive to hydrides; sensor W1C is sensitive to aromatic compounds; sensor W3C is sensitive to amine and aromatic compounds; and sensor W5C is sensitive to olefins, aromatics, and polar molecules.

The volatile components in LFD were identified using electronic nose sensors (Figure 2). Sensors W1W (sensitive to terpenes and organic sulfur compounds), W5S (sensitive to nitrogen compounds), W1S (sensitive to methyl compounds), and W2S (sensitive to alcohol and aldehyde compounds) exhibited higher response values in all LFD samples. This suggests that the volatile aroma components in LFD primarily consist of terpenes, sulfides, nitrogen compounds, methyl compounds, alcohols, and aldehyde ketones (Figure 2a). The Mann–Whitney test showed significant differences in the response values of seven sensors between TLFD and SLFD (Figure 2a,b). The response values of sensors W5S, W5C (sensitive to olefins, aromatics, and polar molecules), W3C (sensitive to amine and aromatic components), and W1C (sensitive to aromatic hydrocarbons) were significantly higher in TLFD (*p* < .05), while those of sensors W1S, W2S, and W3S (sensitive to alkane substances) were significantly higher in SLFD (*p* < .05). The average response values of sensors W1C, W3C, W5C, and W2S (sensitive to alcohol and aldehyde compounds) to TLFD were 0.26, 0.33, 0.34, and 7.72, whereas the response values to SLFD were 0.25, 0.32, 0.33, and 8.04, respectively. Therefore, the aromatic components in TLFD may be higher, while alcohol and aldehyde substances may be lower compared to SLFD.

Principal component analysis (PCA) based on aroma components was used to evaluate the similarity between LFD samples from the Taiyuan and Suizhou regions. PC1 and PC2 explained 67.99% of the total variance. PC1 was composed of the response values from sensors W2S, W3S, W5C, W3C, and W1C, while PC2 was composed of the response values from sensors W1W, W2W, and W5S (Figure 2c). Although there was some overlap in the spatial distribution of the 95% confidence intervals of the TLFD and SLFD samples, the Adonis test still indicated a significant difference between the samples (*p* < .05; Figure 2d). This confirmed the significant differences in aroma characteristics between the LFDs from Taiyuan and Suizhou.

#### 3.1.2 Taste analysis

**FIGURE 3:**
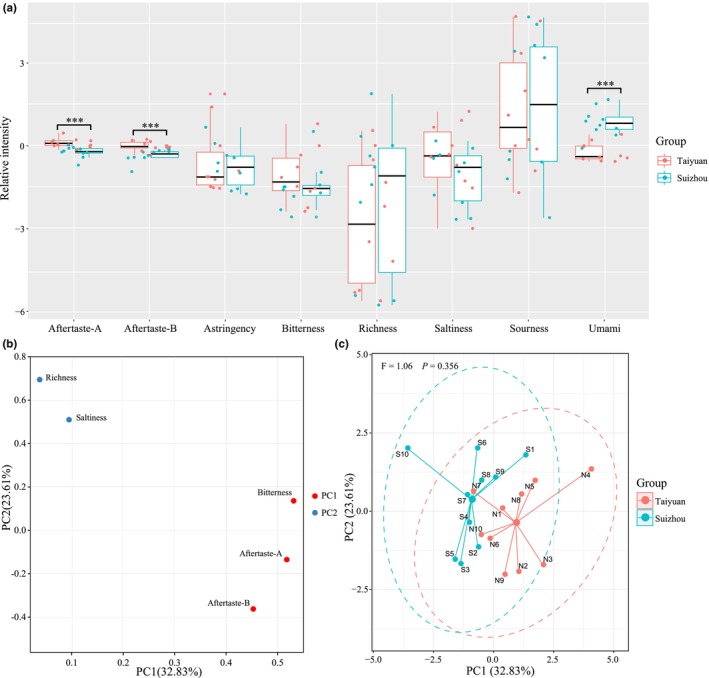
Taste analysis of light‐flavor Daqu based on electronic tongue sensors (a). *** indicates *p* < .001. Principal component analysis loading plot (b) and factor score plot (c) based on taste indicators.

The electronic tongue was used to identify taste indicators in LFD (Figure 3). Both TLFD and SLFD had relatively strong sourness and umami, with an average relative intensity greater than 0. Followed by aftertaste‐A, and aftertaste‐B indicators, with an average relative intensity is at about 0. However, their richness was relatively weak (Figure 3a). The Mann–Whitney test showed that the aftertaste‐A and aftertaste‐B responses were significantly stronger in TLFD than in SLFD, while the umami response was significantly weaker (*p* < .05).

PCA was also used to evaluate the similarity of taste characteristics between LFD samples from both regions. PC1 and PC2 explained 56.44% of the total variance. PC1 was composed of bitterness, aftertaste‐A, and aftertaste‐B, while PC2 was composed of richness and saltiness (Figure 3b). The 95% confidence intervals of TLFD and SLFD samples showed a significant overlap in spatial distribution, and the adonis test indicated that there was no significant difference between them (*p* > .05; Figure 3c). This revealed that the taste characteristics of the TLFD and SLFD samples were comparable.

In addition, when the detection value of a certain indicator changes by more than 1, the taste of the sample will undergo a noticeable change that can be perceived by people (Kobayashi et al., 2010). In this study, the within‐group samples had a range >1 for the five basic tastes (sourness, bitterness, astringency, saltiness, and umami). This indicated that fluctuations in taste quality were more likely to occur compared to odor quality in the production process of LFD.

### 3.2 Microbial diversity analysis

A total of 924,274 bacterial 16S rRNA gene sequences were obtained from the 20 samples, of which 897,185 16S rRNA gene sequences remained after quality control. These effective sequences were classified into 8412 bacterial OTUs based on a sequence similarity of 97%. On average, each sample contained 44,859 effective 16S rRNA gene sequences and 1697 bacterial OTUs. For fungi, after quality control, 1,375,346 effective ITS gene sequences remained out of the 1,379,981 identified ITS gene sequences. These were divided into 1920 fungal OTUs based on a sequence similarity of 97%. On average, each sample contained 68,767 effective ITS gene sequences and 386 fungal OTUs. Observed species increased with increased sequencing depth (Figure S1a,c). For 16S rRNA gene sequences, the dilution curve plateaued when the sequencing reads reached 10,000 (Figure S1b). For ITS2 gene sequences, the dilution curve plateaued when the sequencing reads reached 5000. Hence, the data met the requirements for subsequent bioinformatics analysis in this study (Figure S1d).

**FIGURE 4:**
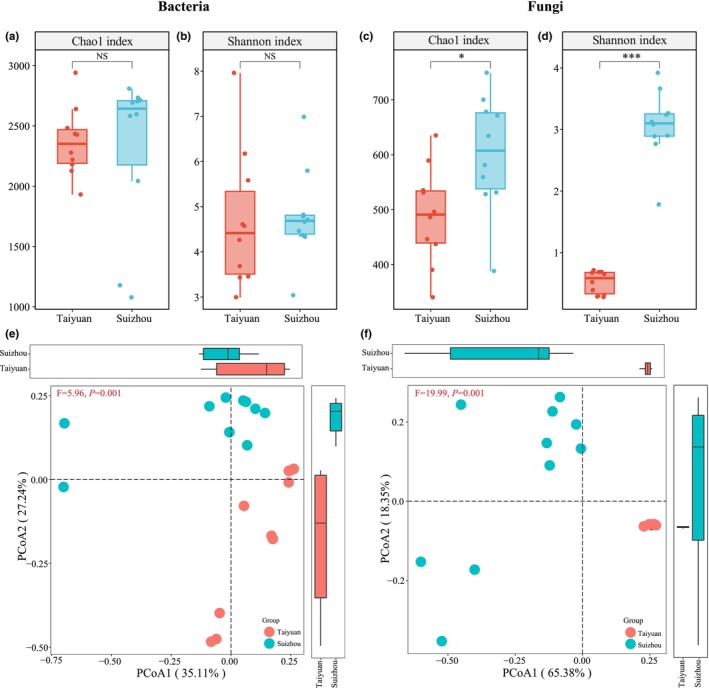
Bacterial Chao1 index (a) and Shannon index (b) of light‐flavor Daqu (LFD) from two regions. Fungal Chao1 index (c) and Shannon index (d) of LFD from two regions. NS indicates *p* > .05; * indicates *p* < .05; *** indicates *p* < .001. Bacterial principal coordinate analysis (PCoA) plots (e) and fungal PCoA plots (f) for LFD from two regions based on Bray–Curtis distances.

The microbial α‐diversity and β‐diversity of TLFD and SLFD samples were comparatively analyzed. The average bacterial Chao1 index values of TLFD and SLFD were 2312 and 2608, respectively, and the average Shannon index values were 4.67 and 4.79, respectively. The difference between the bacterial α‐diversity indices of TLFD and SLFD was not significant (*p* > .05; Figure 4a,b), indicating that bacterial richness and diversity were comparable between LFD samples from both regions. The observed species and Simpson's index were found to be consistent with these results (Figure S2a,c). However, the PCoA plot of spatial distribution based on Bray–Curtis distances revealed a distinct trend of separation between LFD samples from both regions (Figure 4e), and the adonis test showed a significant difference between them (*p* < .05). Hence, the β‐diversity of bacteria was confirmed to be significantly different between LFD samples from the Taiyuan and Suizhou regions.

The average fungal Chao1 index values of TLFD and SLFD were 488 and 601, respectively, and the average Shannon index values were 0.52 and 3.06, respectively. The fungal Chao1 and Shannon indexes of TLFD were significantly lower than those of SLFD (*p* < .05; Figure 4c,d), indicating that the fungal richness and diversity of SLFD were significantly higher than those of TLFD. The observed species and Simpson's index were found to be consistent with these results (Figure S2b,d). Similarly, the PCoA plot based on Bray–Curtis distances showed a separation between LFD samples from the two regions, and the adonis test showed a significant difference between them (*p* < .05; Figure 4f). Hence, the β‐diversity of fungi was confirmed to be significantly different between LFD samples from Taiyuan and Suizhou.

Together, the findings demonstrated the variations in microbial diversity between LFD samples from the Taiyuan and Suizhou regions. Notably, the difference in fungal diversity was more pronounced.

### 3.3 Microbial community analysis

**FIGURE 5:**
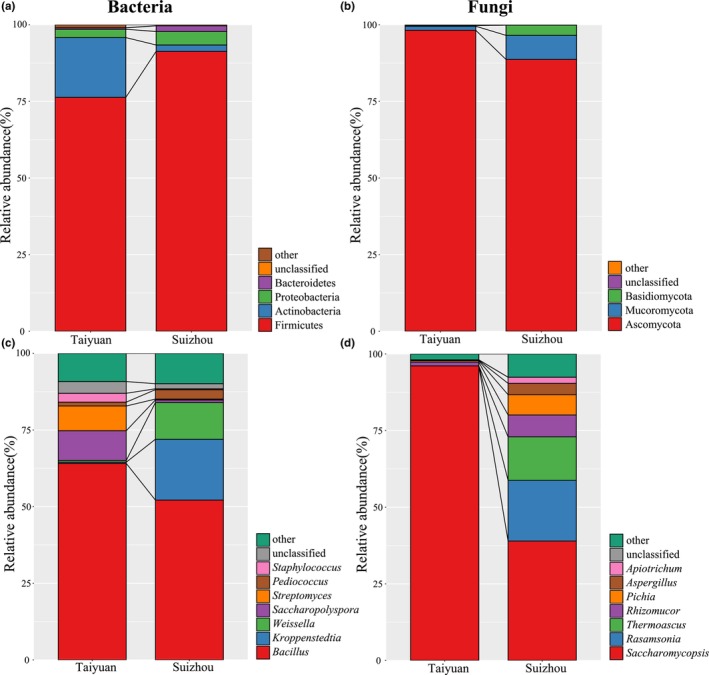
Dominant bacterial phyla (a) and genera (c) in the light‐flavor Daqu (LFD) from two regions. Dominant fungal phyla (b) and genera (d) in the LFD from different regions.

All effective 16S rRNA sequences of 20 samples were annotated to 20 phyla, 53 classes, 82 orders, 158 families, and 310 genera, and the number of unidentified sequences accounted for 0.04% and 2.75% of the total sequences, respectively. In this study, phylums and genera with an average relative abundance of >1.0% were defined as dominant phylums and genera. Four dominant bacterial phyla were found in all samples – Firmicutes (83.18%), Actinobacteria (10.79%), Proteobacteria (3.56%), and Bacteroidetes (1.17%; Figure 5a). The relative abundance of Firmicutes and Bacteroidetes was significantly higher in SLFD, while that of Actinobacteria was significantly lower in TLFD (*p* < .05). Seven dominant bacterial genera were found in all samples – *Bacillus* (58.12%), *Kroppenstedtia* (10.11%), *Weissella* (6.26%), *Saccharopolyspora* (5.27%), *Streptomyces* (4.18%), *Pediococcus* (2.16%), and *Staphylococcus* (1.59%; Figure 5c). The relative abundance of *Saccharopolyspora* and *Staphylococcus* was significantly higher in TLFD, while that of *Kroppenstedtia* was significantly higher in SLFD (*p* < .05).

A total of 6 phyla, 13 classes, 22 orders, 34 families, and 44 genera were annotated from all valid ITS sequences, with unidentified sequences accounting for 0.03% and 0.54% of the total. Three dominant fungal phyla were found in all samples, namely, Ascomycota (88.15%), Mucoromycota (8.23%), and Basidiomycota (3.47%) (Figure 5b). The relative abundance of Ascomycota was significantly higher in TLFD, whereas that of Mucoromycota (8.23%) and Basidiomycota was significantly higher in SLFD (*p* < .05). Seven dominant fungal genera were found in all samples, namely, *Saccharomycopsis* (67.53%), *Rasamsonia* (9.90%), *Thermoascus* (7.10%), *Rhizomucor* (4.17%), *Pichia* (3.53%), *Aspergillus* (1.92%), and *Apiotrichum* (1.09%; Figure 5d). Except for *Saccharomycopsis*, which had a higher relative abundance in TLFD, all other dominant fungal genera showed a higher relative abundance in SLFD (*p* < .05). It is worth noting that only *Saccharomycopsis* and *Rhizomucor* had an average relative abundance >1.0% in TLFD. Therefore, the fungal diversity in TLFD was lower than that in SLFD, consistent with the results of α‐diversity analysis.

**FIGURE 6:**
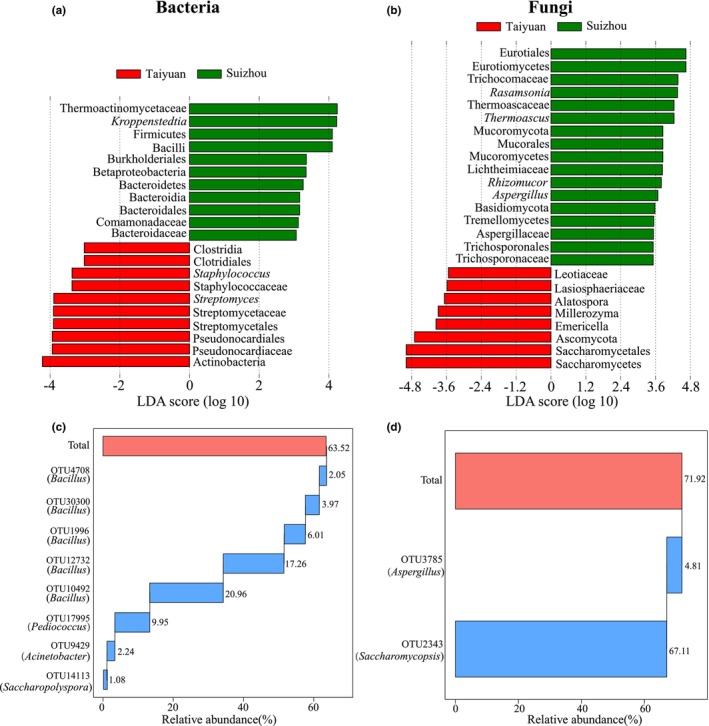
Characteristic bacterial (a) and fungal groups (b) in the light‐flavor Daqu (LFD) from the Taiyuan and Suizhou regions, respectively. Core bacterial (c) and fungal taxa (d) in the LFD from the two regions.

LEfSe analysis was used to identify characteristic microorganisms in the LFD samples from the two regions. When the LDA threshold was set to 3.0, TLFD was found to contain 10 characteristic bacterial groups, with the majority belonging to Actinobacteria and a smaller proportion belonging to Firmicutes. *Streptomyces* and *Staphylococcus* were biomarkers at the genus level (Figure 6a). Meanwhile, SLFD contained 11 characteristic bacterial groups, with the majority belonging to Firmicutes and a smaller proportion belonging to Proteobacteria. *Kroppenstedtia* was a biomarker at the genus level (Figure 6a). When the LDA threshold was set to 4.0, TLFD was found to contain eight characteristic fungal groups, all of which belonged to Ascomycota. *Saccharomycetes* was the fungal group with the highest LDA score, but no biomarkers could be identified at the genus level (Figure 6b). SLFD included 17 characteristic fungal groups, with the majority belonging to Basidiomycota and a smaller proportion belonging to Mucoromycota and Ascomycota. *Thermoascus* and *Aspergillus* were the biomarkers at the genus level (Figure 6b). Therefore, the characteristic fungal groups were more diverse in the LFD from the Suizhou region.

The number of OTUs in each sample was also counted. All samples contained a total of 20 common bacterial OTUs, of which 8 had a relative abundance >1.0% and were defined as the core OTUs. The core bacterial OTUs were OTU4708 (*Bacillus*, 2.05%), OTU30300 (*Bacillus*, 3.97%), OTU1996 (*Bacillus*, 6.01%), OTU12732 (*Bacillus*, 17.26%), OTU10492 (*Bacillus*, 20.96%), OTU17995 (*Pediococcus*, 9.95%), OTU9429 (*Acinetobacter*, 2.24%), and OTU14113 (*Saccharopolyspora*, 1.08%) (Figure 6c). Their cumulative relative abundance was 63.52%. All samples contained a total of 2 core fungal OTUs with a relative abundance >1.0%, namely, OTU3785 (*Aspergillus*, 4.81%) and OTU2343 (*Saccharomycopsis*, 67.11%; Figure 6d). Therefore, *Bacillus*, *Pediococcus*, and *Saccharomycopsis* were found to be the core microbial groups in the LFD samples.

### 3.4 Correlation analysis between dominant microbial taxa and sensory indexes

**FIGURE 7:**
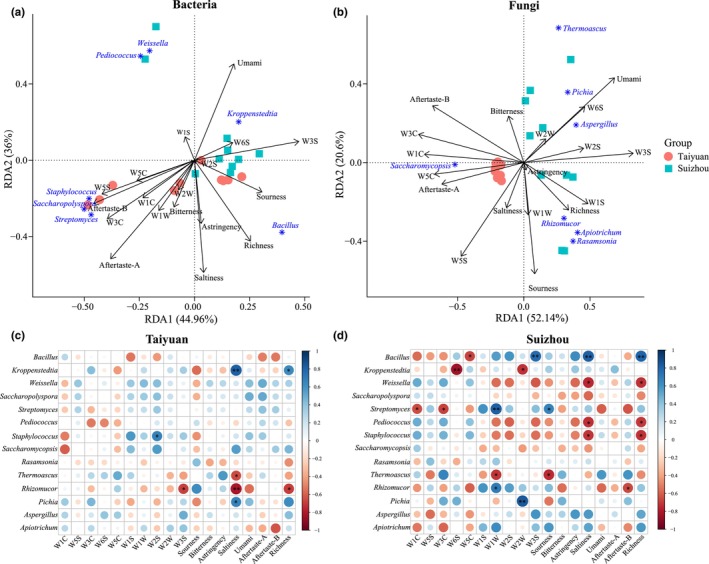
Relationships between dominant bacterial (a) and fungal genera (b) and sensory indicators based on redundancy analysis. Pearson's test was used to analyze the correlations between dominant microbial taxa and their sensory indicators in TLFD samples (c) and SLFD samples (d).

RDA was used to explore the correlations between dominant bacteria and fungi and sensory indicators in all LFD samples. For bacteria, *Staphylococcus*, *Saccharopolyspora*, *Streptomyces*, and *Bacillus* contributed to the variation in the TLFD bacterial community; meanwhile, *Weissella*, *Pediococcus*, and *Kroppenstedtia* contributed to the variation in the SLFD bacterial community (Figure 7a). *Weissella* and *Pediococcus* showed positive correlations with the response value of the W1S sensor, while *Kroppenstedtia* showed positive correlations with umami responses and the response value of the W6S and W3S sensors. *Bacillus* was positively correlated with sourness, richness, astringency, and saltiness. All other indicators were positively correlated with *Staphylococcus*, *Saccharopolyspora*, and *Streptomyces* (Figure 7a).

For fungi, only *Saccharomycopsis* contributed to the variation in the TLFD fungal community, whereas all other dominant fungi contributed to the variation in the fungal community of SLFD (Figure 7b). *Saccharomycopsis* showed positive correlations with saltiness, aftertaste‐A, and the response values of the W5S and W5C sensors. *Rhizomucor*, *Apiotrichum*, and *Rasamsonia* were positively correlated with astringency, richness, and the response values of the W1S and W1W sensors. *Thermoascus*, *Pichia*, and *Aspergillus* were positively correlated with richness and the response values of the W2W, W6S, W2S, and W3S sensors (Figure 7b).

We further explored significant correlations between dominant microbial taxa and sensory indicators using Pearson's test. In TLFD, both *Kroppenstedtia* and *Pichia* were significantly positively correlated with saltiness (*p* < .05), while *Rhizomucor* was significantly negatively correlated with saltiness, richness, and the response value of the W3S sensor (Figure 7c). In addition, there was a significant positive correlation between *Staphylococcus* and the response value of the W2S sensor. Notably, most of the positive correlations were found between dominant bacteria and sensory indicators.

Compared to the TLFD samples, the correlations between dominant microbiota and sensory indicators in SLFD were stronger. In SLFD, there were six bacterial genera (*Bacillus*, *Weissella*, *Pediococcus*, *Staphylococcus*, *Kroppenstedtia*, and *Streptomyces*) and three fungal genera (*Rhizomucor*, *Thermoascus*, and *Rhizomucor*) showing significant correlations with sensory indicators (Figure 7d). *Bacillus* showed significant positive correlations with the response value of the W3S sensor, saltiness, and richness, and negative correlations with the response value of the W5C sensor. *Weissella*, *Pediococcus*, and *Staphylococcus* showed significant negative correlations with the saltiness and umami indicators. *Thermoascus* showed significant negative correlations with sourness and the response value of the W1W sensor. *Streptomyces* exhibited significant negative correlations with the response values of the W1C and W3C sensors and significant positive correlations with sourness and the response value of the W1W sensor. Meanwhile, *Rhizomucor* exhibited a significant positive correlation with the response values of the W1W sensor and a significant negative correlation with the aftertaste‐B indicator. Overall, the correlations between the dominant microbial groups and sensory indicators differed significantly between the LFD from the Taiyuan and Suizhou regions.

## 4 DISCUSSION

LFB originated in Shanxi Province, China, and the flavor of LFB produced in this region is most typical in terms of its clear and pure characteristics. The flavor of LFB from Hubei province is slightly richer, although it retains the typical characteristics of LFB (Xiang et al., 2023). In this study, samples of LFD produced in Taiyuan, Shanxi Province, and Suizhou, Hubei Province, were examined. Electronic sensing technology and high‐throughput sequencing were used to analyze the sensory features and microbial diversity of the LFD from the two regions, and the correlations between these characteristics were evaluated. Accordingly, the study attempted to explain the reasons underlying regional differences in LFB flavor based on Daqu characteristics.

LFD accounts for about 9% of the weight of dry grains used for the production of LFB. Although this percentage is relatively small, the volatile components in LFD are transported into the Jiupei (fermented grains) during the brewing process and are finally collected as part of the liquor following distillation (Wu et al., 2009). Thus, the compounds present in LFD affect the volatile composition of LFB to a certain extent. Hence, it is necessary to analyze the volatile content of LFD. In this study, electronic nose sensor tests showed that the main volatile compounds in LFD samples from both regions were sulfides, nitrogen oxides, methyl compounds, alcohols, aldehydes, and ketones. Unexpectedly, the response values of W5S, W1C, W3C, and W5C sensors were all significantly higher for TLFD. Given that the WC sensors are all sensitive to aromatic compounds, this indicates that TLFD may be richer in aromatic compounds. However, the response values of sensors W1S, W2S, and W3S were all significantly higher for SLFD, indicating that SLFD may be richer in methyl compounds, alcohols, aldehydes, ketones, and long‐chain alkanes. The results of PCA also revealed significant differences in the odor characteristics of the LFD from the two regions. The formation of aroma compounds in Daqu is mainly dependent on the raw materials themselves, microbial metabolites, enzyme‐catalyzed biosynthesis, and non‐enzymatic chemical reactions (Liu & Sun, 2018). The raw materials of LFD are wheat and peas. Peas contain 2‐methyl‐3‐ethylpyrazine and 2‐methoxy‐3‐isobutylpyrazine, which can serve as precursors for the aroma components of Baijiu and give it a strong nutty aroma (Trindler et al., 2022). Therefore, the use of higher‐quality raw materials for the production and brewing of Baijiu can have a positive impact on its quality. Many species from the genera *Bacillus*, *Saccharomyces*, and *Wickerhamomyces* can produce aroma substances and their precursors by metabolizing carbohydrates via enzymatic catalysis (Fan et al., 2019; Tang et al., 2023). However, non‐enzymatic reactions such as thermal degradation contribute to the aroma composition of LFD to a lesser extent due to the low fermentation temperature of LFD (Hou et al., 2022). Thus, the differences in the odor characteristics of LFD from Taiyuan and Suizhou may be the result of differences in the raw materials used and the microbial taxa they contain.

Electronic tongue sensors identified five basic tastes and three aftertastes in the LFD. The LFD from both regions showed the highest response values for sourness, followed by umami, aftertaste‐A, and aftertaste‐B. The weakest intensity was observed for the richness indicator. The prominent sourness of the LFD could be attributed to the lactic acid produced by lactic acid bacteria, as well as the free amino acids and fatty acids produced during microbial decomposition and the utilization of proteins and lipids present in the raw materials (Zhang et al., 2022). TLFD samples showed significantly higher values for the aftertaste‐A and aftertaste‐B indicators, but their umami intensity was lower than that of SLFD samples. However, PCA demonstrated that there were no significant differences in the taste characteristics of the LFD from the two regions. In addition, fluctuations in taste quality were more likely to occur compared to odor quality in the production process of LFD.

The present study further explored the α‐diversity and β‐diversity of microbial communities in the LFD from the two regions. While there were no significant differences between the bacterial abundance and diversity of the two groups, fungal abundance and diversity were significantly lower in TLFD samples than in SLFD samples. PCoA plots showed significant separation trends in spatial arrangement for both bacterial and fungal sample points in TLFD and SLFD. The adonis test yielded a *p*‐value of <.05, indicating that there were significant differences between the microbial community structures of samples from the two regions. Species annotation revealed that the dominant bacterial genera in the LFD of the two regions were *Bacillus*, *Kroppenstedtia*, *Weissella*, *Pediococcus*, and *Staphylococcus* (belonging to Firmicutes) and *Saccharopolyspora* and *Streptomyces* (belonging to Actinobacteria). The dominant fungal genera in the LFD samples were *Saccharomycopsis*, *Rasamsonia*, *Thermoascus*, *Pichia*, and *Aspergillus* (belonging to Ascomycota), *Rhizomucor* (belonging to Mucoromycota), and *Apiotrichum* (belonging to Basidiomycota). The LFDs from the two regions differed significantly in the relative abundance of some dominant microbial communities. Notably, the fungal community was more homogeneous in TLFD than in SLFD. The relative abundance of *Saccharomycopsis* exceeded 95% in TLFD, suggesting that the abundance of molds in TLFD may be low. Molds play a major role in saccharification in LFD. During the Qu‐making process, molds are generally the first group of microbes to show dominance, and they break down large molecules such as starch in the raw material into fermentable sugars such as polysaccharides and monosaccharides. These sugars are easily used and transformed further by bacteria and yeasts, and fermentation proceeds accordingly (Barbagallo et al., 2004; Kaur & Satyanarayana, 2004). A low abundance of molds could lead to weaker saccharification reactions, compromising the growth conditions of other yeasts. This may be one of the reasons for the homogeneous population of dominant yeast genera in TLFD. The Shangmei stage at the beginning of fermentation involves a lower temperature but the highest humidity, and it is the best stage for the growth and multiplication of molds. Thus, in the Taiyuan region, more attention should be given to the Shangmei stage of LFD, and it should be further improved.

LEfSe analysis screened out the characteristic microorganisms in the LFD from both production areas. At the same threshold, there were more than twice as many characteristic fungal communities in SLFD than in TLFD, indicating that fungi were more abundant in SLFD. This is consistent with the results of the α‐diversity analysis. In TLFD samples, the main characteristic bacterial groups were *Streptomyces* and *Staphylococcus*, and the main characteristic fungal group was Saccharomycetes. In SLFD, the main characteristic bacterial group was *Kroppenstedtia*, and the main characteristic fungal groups were *Rasamsonia*, *Thermoascus*, *Aspergillus*, and *Rhizomucor*. The core microbial groups in the LFD from both regions were *Bacillus*, *Pediococcus*, *Saccharomycopsis*, and *Aspergillus*. Previous studies have demonstrated that these core groups are prevalent in Daqu and play an important role in the brewing of Baijiu (Hu et al., 2021; Zhou et al., 2022). *Pediococcus*, as a group of lactic acid bacteria, produces lactic acid, which has an important impact on the aftertaste of Baijiu (Pang et al., 2021; Xu et al., 2020). *Bacillus*, *Saccharomycopsis*, and *Aspergillus* all have a strong ability to break down starch and can also positively impact the production of aroma substances such as ethyl acetate and pyrazines (Fan et al., 2019; Tang et al., 2023; Yang, Wang, et al., 2021).

RDA showed that different microbes affected different sensory indicators of LFD. For example, *Bacillus* was positively correlated with sourness, richness, astringency, and saltiness, and *Saccharomycopsis* was positively correlated with saltiness, aftertaste‐A, and the response value of the W5S and W5C sensors. In addition, *Bacillus*, *Kroppenstedtia*, *Streptomyces*, and *Rhizomucor* showed the highest number of significant correlations with sensory indicators. Previous reports show that both *Kroppenstedtia* and *Rhizomucor* have strong esterification capacity and can enhance the production of ethyl acetate, which confers an aroma important for Baijiu flavor (Chen et al., 2021; Krishna et al., 2001). *Streptomyces* can produce geomycin, the main source of earthy aromas in Baijiu, although too much geomycin can also negatively impact the flavor of Baijiu (Du et al., 2013; Yan et al., 2021). Noteworthily, the correlation between microbiota and sensory indicators was stronger in SLFD, possibly indicating that the microbiota in SLFD produced a richer variety of flavor substances. Although there was a much less significant correlation between microbiota and sensory features in TLFD, TLFD was richer in aromatic substances than SLFD. One of the characteristics of LFB is its pure aroma, and LFB from the Shanxi region is more prominent in this respect (Li et al., 2011). This could be related to the flavor quality of the LFD used in this region.

This study is limited by the lack of research on the sensory features and microbial diversity of the Jiupei. Baijiu is obtained through the distillation of the Jiupei, which serves as the direct source of Baijiu. Consequently, the Jiupei plays an equally important role as the LFD in shaping the flavor of LFB. Subsequently, our future work will focus on analyzing the sensory features and microbial communities in the Jiupei in Taiyuan and Suizhou regions. In conclusion, there are significant differences in flavor characteristics and microbial diversity between the two regions for LFD. Additionally, the correlation between microbial communities and sensory indicators differs between them.

## 5 CONCLUSION

This study conducted a comparative analysis of the sensory and microbial characteristics of LFD from the Taiyuan and Suizhou regions of China and examined the correlations between these features. The findings revealed significant differences in odor, fungal diversity, and the correlation between the microbiota and sensory features in the LFD from the two regions. Although the abundance of the core microorganisms in the LFD from the two regions was large, they still contained various characteristic microorganisms. This research confirmed the impact of different environments and production process differences on the microbial ecology within the LFD, leading to different sensory characteristics. Additionally, this study also found a low mold content in the TLFD, which may indicate that the temperature during the first stage of fermentation (Shangmei stage) is too high, suggesting the need for adjustment of this process parameter. This study established the foundation for a deeper understanding of the microbial composition in the brewing process of LFD while providing scientific support for the control of the production process of Baijiu.

## AUTHOR CONTRIBUTIONS

**Fanshu Xiang:** Resources (equal); visualization (equal); writing – review and editing (equal). **Wenchao Cai:** Formal analysis (equal); methodology (equal); software (equal). **Zhuang Guo:** Data curation (equal); funding acquisition (equal); supervision (equal); visualization (equal). **Chunhui Shan:** Conceptualization (equal); funding acquisition (equal); methodology (equal).

## CONFLICT OF INTEREST STATEMENT

The authors affirm that they have no known financial or interpersonal conflicts that would have appeared to have an impact on the research presented in this paper.

## Supporting information


Figure S1‐S2.

## Data Availability

The data that support the findings of this study are available on request from the corresponding author. The data are not publicly available due to privacy restrictions.

## References

[fsn34004-bib-0001] Barbagallo, R. N. , Spagna, G. , Palmeri, R. , Restuccia, C. , & Giudici, P. (2004). Selection, characterization and comparison of β‐glucosidase from mould and yeasts employable for enological applications. Enzyme and Microbial Technology, 35(1), 58–66. 10.1016/j.enzmictec.2004.03.005

[fsn34004-bib-0002] Brandt, M. I. , Trouche, B. , Quintric, L. , Günther, B. , Wincker, P. , Poulain, J. , & Arnaud‐Haond, S. (2021). Bioinformatic pipelines combining denoising and clustering tools allow for more comprehensive prokaryotic and eukaryotic metabarcoding. Molecular Ecology Resources, 21(6), 1904–1921. 10.1111/1755-0998.13398 33835712

[fsn34004-bib-0003] Cai, W. , Tang, F. , Guo, Z. , Guo, X. , Zhang, Q. , Zhao, X. , Ning, M. , & Shan, C. (2020). Effects of pretreatment methods and leaching methods on jujube wine quality detected by electronic senses and HS‐SPME–GC–MS. Food Chemistry, 330, 127330. 10.1016/j.foodchem.2020.127330 32569941

[fsn34004-bib-0004] Cai, W. , Wang, Y. , Ni, H. , Liu, Z. , Liu, J. , Hou, Q. , Shan, C. , Yang, X. , & Guo, Z. (2021). Diversity of microbiota, microbial functions, and flavor in different types of low‐temperature Daqu. Food Research International, 150, 110734. 10.1016/j.foodres.2021.110734 34865753

[fsn34004-bib-0005] Caporaso, J. G. , Kuczynski, J. , Stombaugh, J. , Bittinger, K. , Bushman, F. D. , Costello, E. K. , Fierer, N. , Peña, A. G. , Goodrich, J. K. , & Gordon, J. I. (2010). QIIME allows analysis of high‐throughput community sequencing data. Nature Methods, 7(5), 335–336. 10.1038/nmeth.f.303 20383131 PMC3156573

[fsn34004-bib-0006] Chen, X. , Huang, J. , Zhou, R. , Zhang, S. , Dong, Y. , Wang, C. , Wang, X. , Wu, C. , & Jin, Y. (2021). Effects of fortifying patterns on the characteristics of strong flavor type Daqu. Food and Fermentation Industries, 47, 50–55.

[fsn34004-bib-0007] Du, H. , Lu, H. , Xu, Y. , & Du, X. (2013). Community of environmental streptomyces related to geosmin development in Chinese liquors. Journal of Agricultural and Food Chemistry, 61(6), 1343–1348. 10.1021/jf3040513 23373536

[fsn34004-bib-0008] Edgar, R. C. , Haas, B. J. , Clemente, J. C. , Quince, C. , & Knight, R. (2011). UCHIME improves sensitivity and speed of chimera detection. Bioinformatics, 27(16), 2194–2200. 10.1093/bioinformatics/btr381 21700674 PMC3150044

[fsn34004-bib-0009] Fan, G. , Teng, C. , Xu, D. , Fu, Z. , Liu, P. , Wu, Q. , Yang, R. , & Li, X. (2019). Improving ethyl acetate production in baijiu manufacture by *Wickerhamomyces anomalus* and *Saccharomyces cerevisiae* mixed culture fermentations. BioMed Research International, 2019, 1–11. 10.1155/2019/1470543 PMC6348840 30733956

[fsn34004-bib-0010] Gan, S.‐H. , Yang, F. , Sahu, S. K. , Luo, R.‐Y. , Liao, S.‐L. , Wang, H.‐Y. , Jin, T. , Wang, L. , Zhang, P.‐F. , & Liu, X. (2019). Deciphering the composition and functional profile of the microbial communities in Chinese Moutai liquor starters. Frontiers in Microbiology, 10, 1540. 10.3389/fmicb.2019.01540 31333631 PMC6620787

[fsn34004-bib-0011] Hong, J. , Tian, W. , & Zhao, D. (2020). Research progress of trace components in sesame‐aroma type of baijiu. Food Research International, 137, 109695. 10.1016/j.foodres.2020.109695 33233269

[fsn34004-bib-0012] Hong, J. , Zhao, D. , & Sun, B. (2021). Research progress on the profile of trace components in baijiu. Food Reviews International, 39, 1666–1693. 10.1080/87559129.2021.1936001

[fsn34004-bib-0013] Hou, Q. , Wang, Y. , Cai, W. , Ni, H. , Zhao, H. , Zhang, Z. , Liu, Z. , Liu, J. , & Guo, Z. (2022). Metagenomic and physicochemical analyses reveal microbial community and functional differences between three types of low‐temperature Daqu. Food Research International, 156, 111167. 10.1016/j.foodres.2022.111167 35651033

[fsn34004-bib-0014] Hu, X. , Wang, K. , Chen, M. , Fan, J. , Han, S. , Hou, J. , Chi, L. , Liu, Y. , & Wei, T. (2020). Profiling the composition and metabolic activities of microbial community in fermented grain for the Chinese strong‐flavor baijiu production by using the metatranscriptome, high‐throughput 16S rRNA and ITS gene sequencings. Food Research International, 138, 109765. 10.1016/j.foodres.2020.109765 33292946

[fsn34004-bib-0015] Hu, Y. , Huang, X. , Yang, B. , Zhang, X. , Han, Y. , Chen, X.‐X. , & Han, B.‐Z. (2021). Contrasting the microbial community and metabolic profile of three types of light‐flavor Daqu. Food Bioscience, 44, 101395. 10.1016/j.fbio.2021.101395

[fsn34004-bib-0016] Huang, Z.‐R. , Guo, W.‐L. , Zhou, W.‐B. , Li, L. , Xu, J.‐X. , Hong, J.‐L. , Liu, H.‐P. , Zeng, F. , Bai, W.‐D. , & Liu, B. (2019). Microbial communities and volatile metabolites in different traditional fermentation starters used for Hong Qu glutinous rice wine. Food Research International, 121, 593–603. 10.1016/j.foodres.2018.12.024 31108786

[fsn34004-bib-0017] Karakaya, D. , Ulucan, O. , & Turkan, M. (2020). Electronic nose and its applications: A survey. International Journal of Automation and Computing, 17(2), 179–209. 10.1007/s11633-019-1212-9

[fsn34004-bib-0018] Kaur, P. , & Satyanarayana, T. (2004). Production and starch saccharification by a thermostable and neutral glucoamylase of a thermophilic mould *Thermomucor indicae‐seudaticae* . World Journal of Microbiology and Biotechnology, 20, 419–425. 10.1023/B:WIBI.0000033065.22647.5b

[fsn34004-bib-0019] Kobayashi, Y. , Habara, M. , Ikezazki, H. , Chen, R. , Naito, Y. , & Toko, K. (2010). Advanced taste sensors based on artificial lipids with global selectivity to basic taste qualities and high correlation to sensory scores. Sensors, 10(4), 3411–3443. 10.3390/s100403411 22319306 PMC3274227

[fsn34004-bib-0020] Krishna, S. H. , Divakar, S. , Prapulla, S. , & Karanth, N. (2001). Enzymatic synthesis of isoamyl acetate using immobilized lipase from *Rhizomucor miehei* . Journal of Biotechnology, 87(3), 193–201. 10.1016/S0168-1656(00)00432-6 11334663

[fsn34004-bib-0021] Li, Z. , Wang, N. , Raghavan, G. , & Vigneault, C. (2011). Volatiles evaluation and dielectric properties measurements of Chinese spirits for quality assessment. Food and Bioprocess Technology, 4(2), 247–253. 10.1007/s11947-008-0162-y

[fsn34004-bib-0022] Liu, H. , & Sun, B. (2018). Effect of fermentation processing on the flavor of baijiu. Journal of Agricultural and Food Chemistry, 66(22), 5425–5432. 10.1021/acs.jafc.8b00692 29751730

[fsn34004-bib-0023] Nilsson, R. H. , Larsson, K.‐H. , Taylor, A. F. S. , Bengtsson‐Palme, J. , Jeppesen, T. S. , Schigel, D. , Kennedy, P. , Picard, K. , Glöckner, F. O. , & Tedersoo, L. (2019). The UNITE database for molecular identification of fungi: Handling dark taxa and parallel taxonomic classifications. Nucleic Acids Research, 47(D1), D259–D264. 10.1093/nar/gky1022 30371820 PMC6324048

[fsn34004-bib-0024] Pang, X.‐N. , Chen, C. , Huang, X.‐N. , Yan, Y.‐Z. , Chen, J.‐Y. , & Han, B.‐Z. (2021). Influence of indigenous lactic acid bacteria on the volatile flavor profile of light‐flavor baijiu. LWT, 147, 111540. 10.1016/j.lwt.2021.111540

[fsn34004-bib-0025] Ross, C. F. (2021). Considerations of the use of the electronic tongue in sensory science. Current Opinion in Food Science, 40, 87–93. 10.1016/j.cofs.2021.01.011

[fsn34004-bib-0026] Sun, X. , Lyu, G. , Luan, Y. , Zhao, Z. , Yang, H. , & Su, D. (2018). Analyses of microbial community of naturally homemade soybean pastes in Liaoning Province of China by Illumina Miseq sequencing. Food Research International, 111, 50–57. 10.1016/j.foodres.2018.05.006 30007713

[fsn34004-bib-0027] Tang, Q. , Chen, X. , Huang, J. , Zhang, S. , Qin, H. , Dong, Y. , Wang, C. , Wang, X. , Wu, C. , & Jin, Y. (2023). Mechanism of enhancing pyrazines in Daqu via inoculating *bacillus licheniformis* with strains specificity. Food, 12(2), 304. 10.3390/foods12020304 PMC9858619 36673396

[fsn34004-bib-0028] Trindler, C. , Kopf‐Bolanz, K. A. , & Denkel, C. (2022). Aroma of peas, its constituents and reduction strategies–effects from breeding to processing. Food Chemistry, 376, 131892. 10.1016/j.foodchem.2021.131892 34971885

[fsn34004-bib-0029] Wang, W. , Xu, Y. , Huang, H. , Pang, Z. , Fu, Z. , Niu, J. , Zhang, C. , Li, W. , Li, X. , & Sun, B. (2021). Correlation between microbial communities and flavor compounds during the fifth and sixth rounds of sauce‐flavor baijiu fermentation. Food Research International, 150, 110741. 10.1016/j.foodres.2021.110741 34865760

[fsn34004-bib-0030] Wei, Z.‐G. , Zhang, X.‐D. , Cao, M. , Liu, F. , Qian, Y. , & Zhang, S.‐W. (2021). Comparison of methods for picking the operational taxonomic units from amplicon sequences. Frontiers in Microbiology, 12, 644012. 10.3389/fmicb.2021.644012 33841367 PMC8024490

[fsn34004-bib-0031] Wu, X.‐H. , Zheng, X.‐W. , Han, B.‐Z. , Vervoort, J. , & Nout, M. R. (2009). Characterization of Chinese liquor starter,“Daqu”, by flavor type with 1H NMR‐based nontargeted analysis. Journal of Agricultural and Food Chemistry, 57(23), 11354–11359. 10.1021/jf902881p 19886664

[fsn34004-bib-0032] Xiang, F. , Cai, W. , Hou, Q. , Gai, J. , Dong, X. , Li, L. , Liu, Z. , Tian, X. , Shan, C. , & Guo, Z. (2023). Comparative analysis of the microbial community structure in light‐flavor Daqu in Taiyuan and Suizhou regions, China. Lwt, 177, 114599. 10.1016/j.lwt.2023.114599

[fsn34004-bib-0033] Xiao, M. , Xiong, T. , Peng, Z. , Liu, C. , Huang, T. , Yu, H. , & Xie, M. (2018). Correlation between microbiota and flavours in fermentation of Chinese Sichuan Paocai. Food Research International, 114, 123–132. 10.1016/j.foodres.2018.06.051 30361008

[fsn34004-bib-0034] Xu, Y. , Sun, B. , Fan, G. , Teng, C. , Xiong, K. , Zhu, Y. , Li, J. , & Li, X. (2017). The brewing process and microbial diversity of strong flavour Chinese spirits: A review. Journal of the Institute of Brewing, 123(1), 5–12. 10.1002/jib.404

[fsn34004-bib-0035] Xu, Z. , Luo, Y. , Mao, Y. , Peng, R. , Chen, J. , Soteyome, T. , Bai, C. , Chen, L. , Liang, Y. , & Su, J. (2020). Spoilage lactic acid bacteria in the brewing industry. Journal of Microbiology and Biotechnology, 30(7), 955–961. 10.4014/jmb.1908.08069 31986245 PMC9728350

[fsn34004-bib-0036] Yan, Q. , Zhang, K. , Zou, W. , & Hou, Y. (2021). Three main flavour types of Chinese baijiu: Characteristics, research, and perspectives. Journal of the Institute of Brewing, 127(4), 317–326. 10.1002/jib.669

[fsn34004-bib-0037] Yang, C. , You, L. , Kwok, L.‐Y. , Jin, H. , Peng, J. , Zhao, Z. , & Sun, Z. (2021). Strain‐level multiomics analysis reveals significant variation in cheeses from different regions. Lwt, 151, 112043. 10.1016/j.lwt.2021.112043

[fsn34004-bib-0038] Yang, Y. , Fan, Y. , Li, T. , Yang, Y. , Zeng, F. , Wang, H. , Suo, H. , Song, J. , & Zhang, Y. (2022). Microbial composition and correlation between microbiota and quality‐related physiochemical characteristics in chongqing radish paocai. Food Chemistry, 369, 130897. 34455330 10.1016/j.foodchem.2021.130897

[fsn34004-bib-0039] Yang, Y. , Wang, S.‐T. , Lu, Z.‐M. , Zhang, X.‐J. , Chai, L.‐J. , Shen, C.‐H. , Shi, J.‐S. , & Xu, Z.‐H. (2021). Metagenomics unveils microbial roles involved in metabolic network of flavor development in medium‐temperature daqu starter. Food Research International, 140, 110037. 10.1016/j.foodres.2020.110037 33648263

[fsn34004-bib-0040] Zhang, Q. , Shi, J. , Wang, Y. , Zhu, T. , Huang, M. , Ye, H. , Wei, J. , Wu, J. , Sun, J. , & Li, H. (2022). Research on interaction regularities and mechanisms between lactic acid and aroma compounds of baijiu. Food Chemistry, 397, 133765. 10.1016/j.foodchem.2022.133765 35905622

[fsn34004-bib-0041] Zheng, X.‐W. , & Han, B.‐Z. (2016). Baijiu (白酒), Chinese liquor: History, classification and manufacture. Journal of Ethnic Foods, 3(1), 19–25. 10.1016/j.jef.2016.03.001

[fsn34004-bib-0042] Zheng, X.‐W. , Yan, Z. , Nout, M. R. , Smid, E. J. , Zwietering, M. H. , Boekhout, T. , Han, J.‐S. , & Han, B.‐Z. (2014). Microbiota dynamics related to environmental conditions during the fermentative production of fen‐Daqu, a Chinese industrial fermentation starter. International Journal of Food Microbiology, 182, 57–62. 10.1016/j.ijfoodmicro.2014.05.008 24863368

[fsn34004-bib-0043] Zhou, Q. , Ma, K. , Song, Y. , Wang, Z. , Fu, Z. , Wang, Y. , Zhang, X. , Cui, M. , Tang, N. , & Xing, X. (2022). Exploring the diversity of the fungal community in Chinese traditional baijiu Daqu starters made at low‐, medium‐and high‐temperatures. Lwt, 162, 113408. 10.1016/j.lwt.2022.113408

[fsn34004-bib-0044] Zhu, M. , Zheng, J. , Xie, J. , Zhao, D. , Qiao, Z.‐W. , Huang, D. , & Luo, H.‐B. (2022). Effects of environmental factors on the microbial community changes during medium‐high temperature Daqu manufacturing. Food Research International, 153, 110955. 10.1016/j.foodres.2022.110955 35227477

