# Supplementary material for: Comparative analysis of sensory features, microbial diversity, and their correlations in light‐flavor Daqu from different regions
Source: Food Sci Nutr. 2024 Feb 6;12(5):3391–404. doi: 10.1002/fsn3.4004 (PMC11077209; doi:10.1002/fsn3.4004)
Supplement: Supplementary file 1 — Figure S1‐S2. [file FSN3-12-3391-s001.docx]

**Supplementary material for**

**Comparative analysis of sensory features, microbial diversity, and their correlations in light-flavor Daqu from different regions**

**Figure captions**

**Figure S1:** Bacterial observed speices (a) and Simpson index (b) of LFD from two regions. Fungal observed speices (c) and Simpson index (d) of LFD from two regions. NS indicates *P*>0.05; * indicates *P*<0.05; *** indicates *P*<0.001.

**Figure S2:** Rarefaction curves of the bacterial observed species index (a) and Shannon diversity index (b) in LFD samples. The sequencing depth was 28,010 reads. Rarefaction curves of the fungal observed species index (c) and Shannon diversity index (d) for the LFD. The sequencing depth was 50,010 reads.


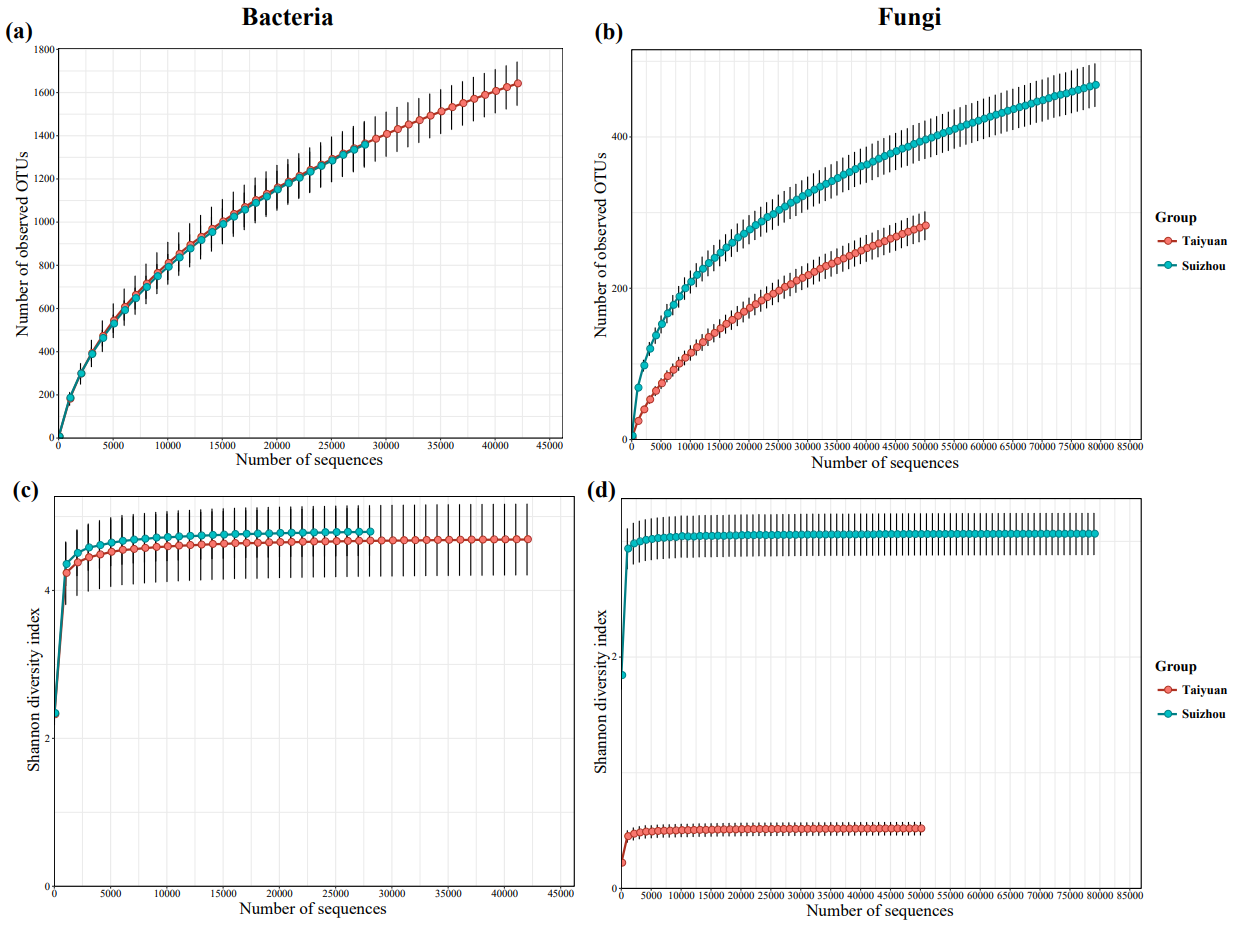


**Figure S1:** Bacterial observed speices (a) and Simpson index (c) of LFD from two regions. Fungal observed speices (b) and Simpson index (d) of LFD from two regions. NS indicates *P*>0.05; * indicates *P*<0.05; *** indicates *P*<0.001.


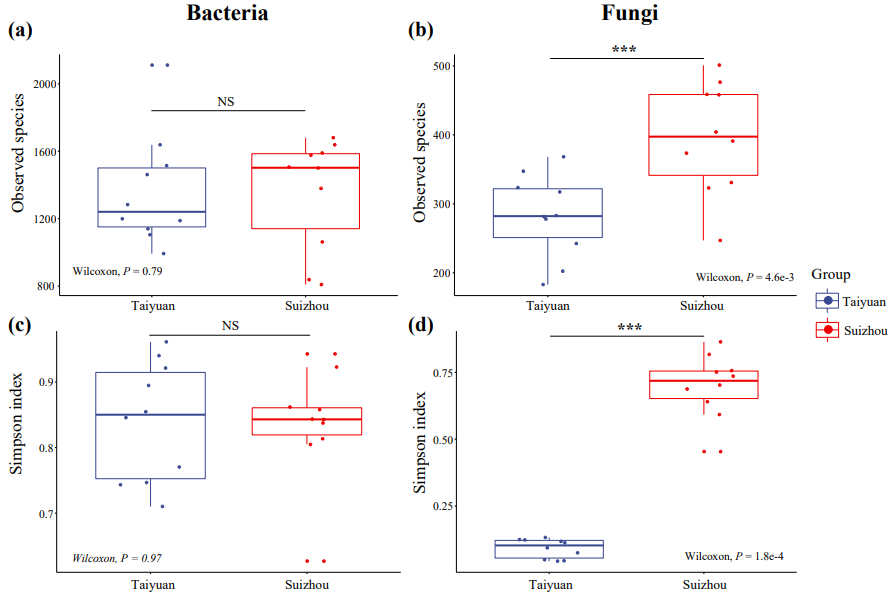


**Figure S2:** Rarefaction curves of the bacterial observed species index (a) and Shannon diversity index (c) in LFD samples. The sequencing depth was 28,010 reads. Rarefaction curves of the fungal observed species index (b) and Shannon diversity index (d) for the LFD. The sequencing depth was 50,010 reads.
